# A cellular senescence-related signature for predicting prognosis, immunotherapy response, and candidate drugs in patients treated with transarterial chemoembolization (TACE)

**DOI:** 10.1007/s12672-024-01116-8

**Published:** 2024-07-08

**Authors:** Ning He, Wenjing Zhao, Wenlong Tian, Ying Wu, Jian Xu, Yunyan Lu, Xudong Chen, Hui Zhao

**Affiliations:** 1 Department of Interventional and Vascular Surgery, Affiliated Hospital of Nantong University, Nantong, China; 2 Cancer Research Center Nantong, Affiliated Tumor Hospital of Nantong University, Nantong, China; 3 Department of Oncology, The Second People’s Hospital of Nantong, Nantong, China; 4 Department of Gynecology, Affiliated Tumor Hospital of Nantong University, Nantong, China; 5 Department of Pathology, Affiliated Tumor Hospital of Nantong University, Nantong, China

**Keywords:** Hepatocellular carcinoma, Transcatheter arterial chemoembolization, Cellular senescence, Prognosis, Tumor immune microenvironment, Immunotherapy

## Abstract

**Background:**

Cellular senescence is essential to TME development, progression, and remodeling. Few studies have examined cellular senescence in HCC after TACE. Investigating the relationship between cellular senescence, post-TACE prognosis, the TME, and immune treatment responses is crucial.

**Methods:**

We analyzed the GSE104580 dataset to identify DEGs. A cellular senescence-related signature was developed using LASSO Cox regression in the GSE14520 dataset and validated in the ICGC dataset. High- and low-risk subgroups were compared using GSVA and GSEA. Correlation studies were conducted to explore the relationship between the prognostic model, immune infiltration, immunotherapy response, and drug sensitivity.

**Results:**

A cellular senescence-related signature comprising FOXM1, CDK1, CHEK1, and SERPINE1 was created and validated. High-risk patients showed significantly lower OS than low-risk patients. High-risk patients had carcinogenetic pathways activated, immunosuppressive cells infiltrated, and immunomodulatory genes overexpressed. They also showed higher sensitivity to EPZ004777_1237 and MK-2206_1053 and potential benefits from GSK-3 inhibitor IX, nortriptyline, lestaurtinib, and JNK-9L.

**Conclusions:**

This study constructed a cellular senescence-related signature that could be used to predict HCC patients’ responses to and prognosis after TACE treatment, aiding in the development of personalized treatment plans.

**Supplementary Information:**

The online version contains supplementary material available at 10.1007/s12672-024-01116-8.

## Introduction

Hepatocellular carcinoma (HCC) is one of the most common malignant tumors, ranking fifth in terms of incidence and third in terms of mortality worldwide [1]. However, due to the invasive growth of the tumor and late symptom manifestation, most HCC patients are diagnosed at an advanced stage, thus missing the window for curative surgery [2]. The Barcelona Clinic Liver Cancer (BCLC) staging system recommends transarterial chemoembolization (TACE) as a first-line treatment for intermediate-stage inoperable HCC [3]. The primary mechanism of TACE combines the direct cytotoxic effects of intra-arterial chemotherapy with ischemic damage caused by stopping blood flow, leading to tumor regression [4]. TACE combines cytotoxic chemotherapy with ischemic damage to induce tumor regression. However, the response rate to initial TACE treatment is less than 40%, and repeat treatments can lead to liver function deterioration [5, 6]. Moreover, excessive use of TACE may offset the potential benefits of effective systemic treatments. Data suggest that less than 20% of TACE nonresponders undergo systemic treatments [7]. Therefore, identifying new signature to predict TACE response and guide treatment strategies is crucial.

Numerous factors contribute to TACE nonresponse, primarily including tumor angiogenesis, the development of chemotherapeutic drug resistance and the tumor immune microenvironment (TIME) [8, 9]. Growing evidence suggests that TACE nonresponse is associated with TIME. Studies have shown that after TACE, the gene expression levels related to the exhaustion, costimulation, and cytotoxic characteristics of CD8 + T cells with robust antitumor activity significantly decrease, while the elevation of tumor-associated macrophages leads to marked immune suppression and promotes tumor angiogenesis, thus restoring tumor blood supply and resulting in TACE nonresponse [10]. Additionally, research indicates that the expression of programmed death-1 (PD1) in TACE-HCC tumor cells is significantly higher than that in non-TACE-HCC, and in resected TACE tumors, the expression of both PD-1 and programmed death ligand-1 (PD-L1) in intratumoral inflammatory cells and tumor cells was significantly elevated compared with that in corresponding pre-TACE biopsies [11].

Cellular senescence, a physiological state of cell cycle arrest under endogenous and exogenous stress, is an important way to control tumour progression and inhibit cancer cell proliferation [12–14]. Many therapeutic drugs induce cellular senescence, such as doxorubicin and cisplatin, which effectively suppresses tumor development [15, 16]. Low-dose chemotherapy induces senescence by causing DNA damage and activating the ATM/ATR-mediated DNA damage response, which in turn activates the p53/p21 and RB pathways, blocking cell division [17, 18]. Some antitumor effects of these chemotherapeutic drugs might depend on senescence-mediated antitumor immunity [19]. However, continuous use of chemotherapeutic drugs can lead to immune suppression within the tumor and cause inflammation and fibrosis in normal tissues [20]. Furthermore, when senescent cells are not cleared by activated immune cells and accumulate in the late stages, the unique SASP of maladaptive senescence enhances tumorigenesis by activating immune suppression, promoting cell proliferation, driving tumor angiogenesis, and advancing tumor progression via epithelial-mesenchymal transition (EMT), angiogenesis, and extracellular matrix degradation signaling [15, 21]. This dual role of senescence in cancer therapy highlights the need for a deeper understanding of its impact on TACE nonresponse and immune modulation in HCC.

Whether TACE nonresponsiveness leads to cellular senescence, subsequently driving tumor progression, remains unclear. In this study, we first investigate the relationship between TACE nonresponsiveness and cellular senescence. Then further screened senescence-related genes and established a risk model for predicting post-TACE prognosis. Finally, based on the risk model, we focused on analyzing the landscape of risk subgroups with tumor staging, immune infiltration and evasion, immune treatment response, and potential therapeutic drugs. Our study provides new insights into the potential mechanisms of post-TACE nonresponsiveness and predictions for immunotherapy in HCC patients.

## Results

### The cellular senescence pathway was enriched in the TACE nonresponse group

**Fig. 1:**
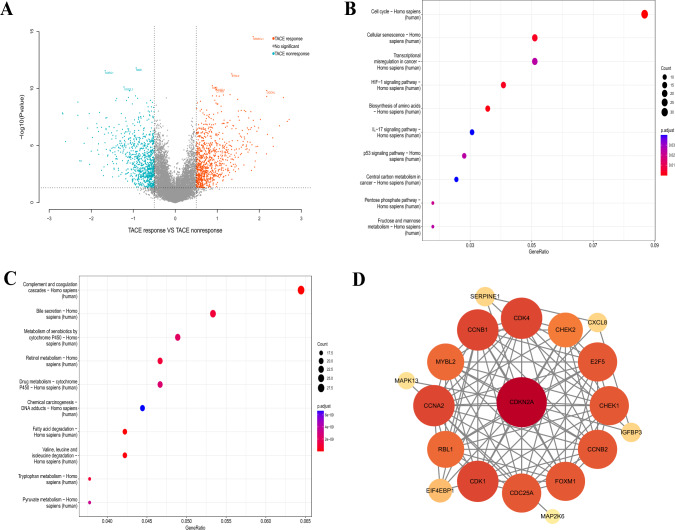
Differences in transcriptome samples between the TACE response and nonresponse groups in GSE104580. **a** The volcano map shows genes upregulated in both groups. The bubble diagram shows KEGG pathways enriched in the TACE (**b**) nonresponse and (**c**) response groups. **d** A PPI of these eight molecules was plotted using Cytoscape

By analyzing differences in gene expression between 81 TACE nonresponders and 66 TACE responders in GSE104580, we identified 859 genes that were upregulated in nonresponders and 875 that were upregulated in responders (Fig. 1a). KEGG enrichment analysis revealed that the cellular senescence pathway was highly enriched among TACE nonresponders (Fig. 1b), but not in TACE responders (Fig. 1c).A protein‒protein interaction (PPI) network was constructed using 20 genes enriched in the cellular senescence pathway (Fig. 1d).

### Identification of cellular senescence pathway-related genes and construction of the prognostic signature

**Fig. 2:**
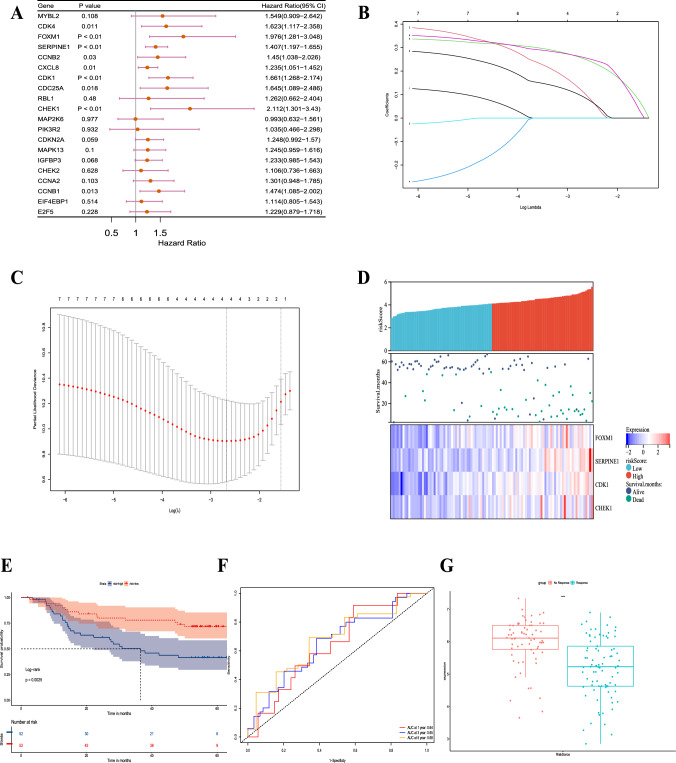
Identification of cellular senescence pathway-related genes and construction of the prognostic signature. **a** The forest map in the table shows that 9 of the 20 genes were related to poor OS in patients treated with TACE. HR: hazard ratio; CI: confidence interval. **b** LASSO coefficient profiles of the nine genes associated with poor prognosis of patients treated with TACE. The coefficient of four genes was not 0 in GSE14520. **c** A coefficient profile plot was generated against the log (λ) sequence. **d** Risk scores of patients treated with TACE in GSE14520 and median risk score. **e** The K–M curve shows that OS was lower in the high-risk group than in the low-risk group in patients treated with TACE in GSE14520. **f** The area under the curve (AUC) value of the time-dependent receiver operating characteristic (ROC) curve shows the prognostic performance of the signature in GSE14520. **g** Difference in risk score between TACE response and nonresponse groups in GSE104580

To construct prognostic markers associated with post-TACE outcomes, we performed univariate regression analysis on the GSE14520 dataset and identified nine genes significantly associated with poor prognosis (Fig. 2a).Using LASSO regression analysis, we refined this list to four genes to create a prognostic signature (Fig. 2b and c). The risk score calculation formula is: Risk score = FOXM1 × 0.108 + CDK1 × 0.258 + CHEK1 × 0.089 + SERPINE1 × 0.252.

Based on the median risk score, we divided the patients into a high-risk group (n = 52) and a low-risk group (n = 52) (Fig. 2d). Kaplan‒Meier analysis showed that the high-risk group had a significantly worse prognosis than the low-risk group (Fig. 2e; P = 0.0025). Time-dependent receiver operating characteristic (ROC) curves demonstrated our signature’s capacity to forecast the outcome in TACE patients. The areas under the curve (AUCs) at 1, 3, and 5 years were 0.64, 0.65, and 0.68, respectively, indicating the good predictive ability of the prognostic features (Fig. 2f). Next, we applied the same formula to calculate the risk scores of samples in the GSE104580 dataset to detect any differences between nonresponders and responders to TACE. As shown in Fig. 2g, the risk score was higher in the nonresponse group than in the responsive group (P < 0.0001).

### The senescence-related signature was specific to TACE

**Fig. 3:**
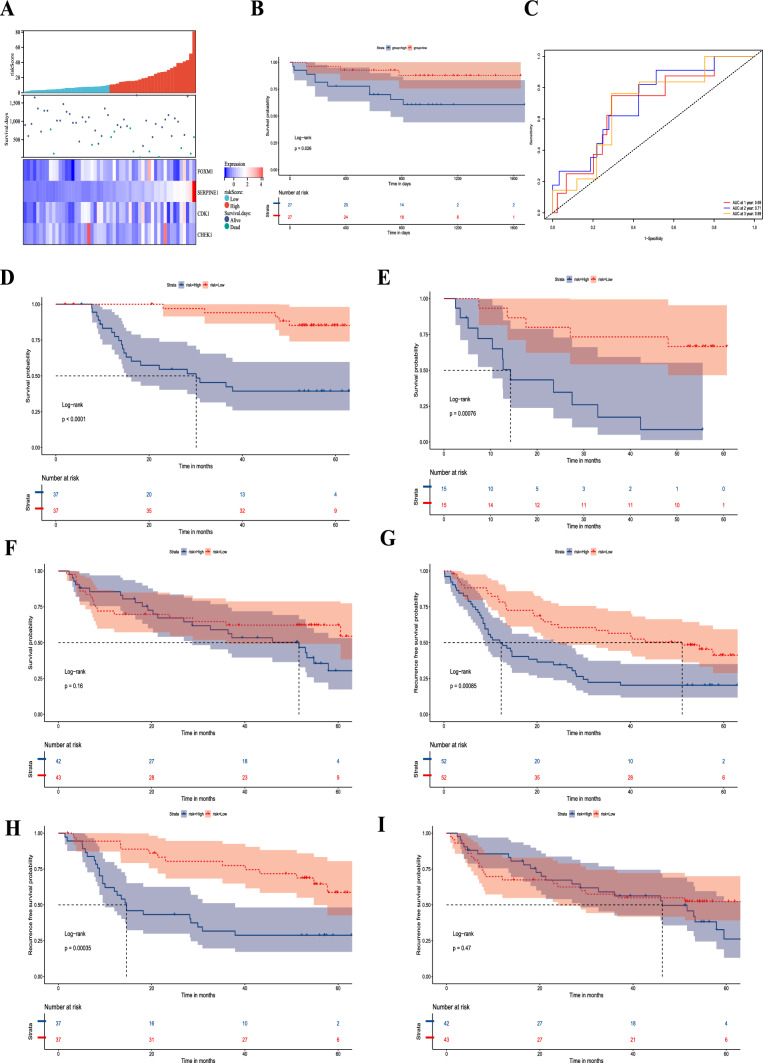
Verification of the signature in ICGC and GSE14520. **a** Risk scores of patients treated with TACE in ICGC and median risk score. **b** The K–M curve shows that OS was lower in the high-risk group than in the low-risk group in patients treated with TACE in ICGC. **c** The area under the curve (AUC) value of the time-dependent receiver operating characteristic (ROC) curve shows the prognostic performance of the signature in ICGC. Survival analysis of patients with different treatment methods. OS of patients treated with (**d**) adjuvant TACE, (**e**) postrecurrence TACE, and (**f**) resection only. Recurrence-free survival (RFS) of patients treated with (**g**) postrecurrence or adjuvant TACE, (**h**) adjuvant TACE, and (**i**) resection only

To verify this signature, we first divided a validation cohort of 54 TACE samples into high-risk and low-risk groups based on the median risk score (Fig. 3a). Kaplan‒Meier analysis showed a significantly better prognosis in the low-risk group than in the high-risk group (Fig. 3b, P = 0.026). ROC analysis showed that the AUCs of 1-, 2-, and 3-year survival were 0.69, 0.71, and 0.69, respectively (Fig. 3c).

Then, to further evaluate whether this signature is specific to patients undergoing TACE treatment and not applicable to all HCC patients, we divided patients treated with postrecurrence TACE, adjuvant TACE, and resection only into high-risk and low-risk groups based on the median risk score in the GSE14520 dataset. Among 104 patients, 74 received adjuvant TACE treatment, and 30 received TACE treatment after recurrence. Kaplan‒Meier analysis showed that among patients with adjuvant TACE treatment (Fig. 3d) and those receiving postrecurrence TACE treatment (Fig. 3e), the overall survival (OS) of the high-risk group was lower. However, we found no difference in patient resection only (Fig. 3f). Moreover, we analyzed recurrence-free survival (RFS) among patients other than those treated with postrecurrence TACE since the recurrence time was measured from resection to recurrence. As expected, among patients receiving TACE treatment (Fig. 3g) and those receiving adjuvant TACE treatment (Fig. 3h), the RFS of the high-risk group was lower. Patients who underwent only surgical resection still showed no difference (Fig. 3i).

### Verification of the signature’s independent prognostic and distinguishing ability in different clinical subgroups

**Fig. 4:**
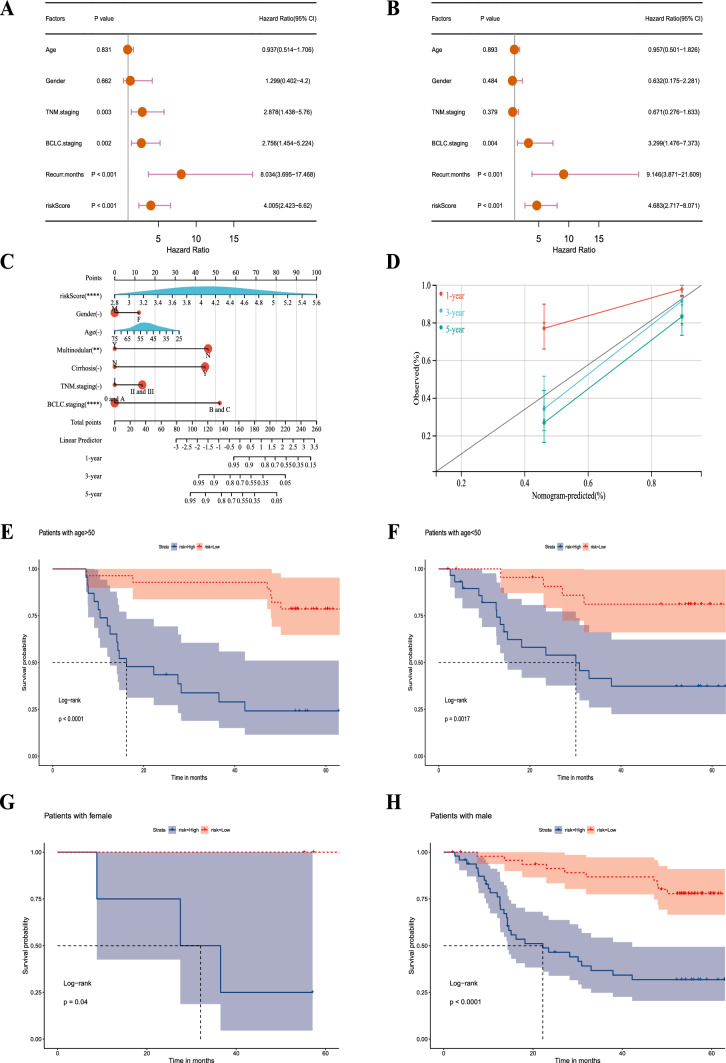

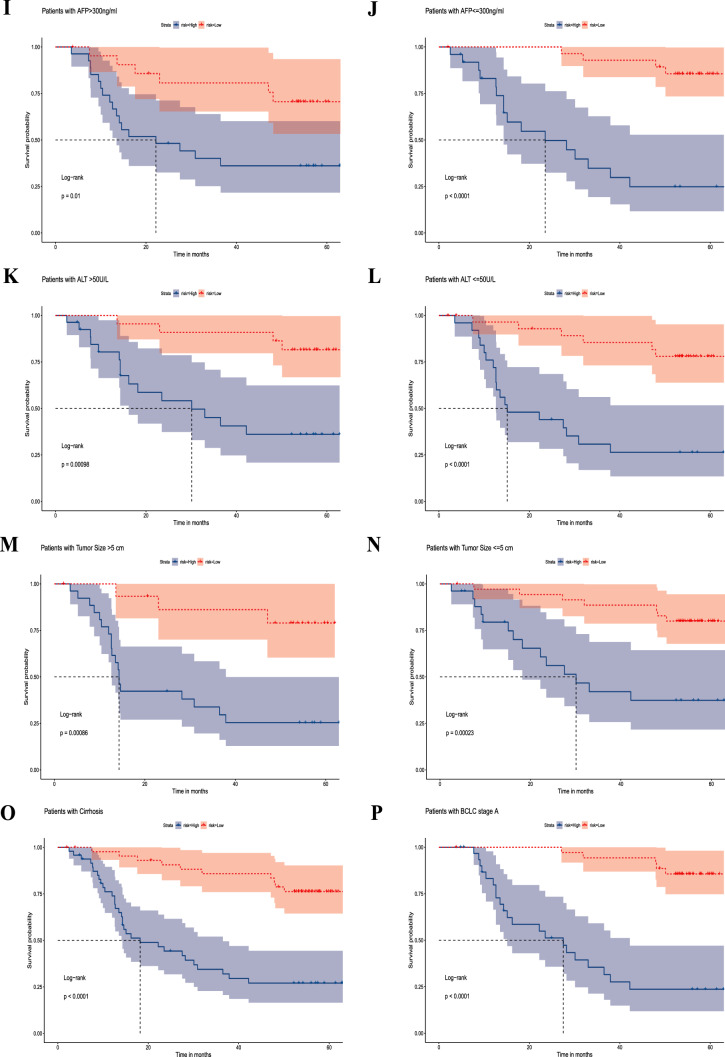
Independent prognostic analysis and clinical correlation analysis of the senescence-related signature. **a** Univariate Cox regression analysis for GSE14520. **b** Multivariate Cox regression analysis for GSE14520. **c** Construction of the nomograms. **d** The calibration curves displaying the accuracy of the nomogram at 1, 3 and 5 years. **e–p** K–M curves for differences in OS among different clinical subgroups of patients in GSE14520 treated with TACE (age ≤ 50 and > 50; female, male, AFP > / ≤ 300 ng/ml, ALT > / ≤ 50 U/L, tumor size > / ≤ 5 cm, cirrhosis and BCLC stage A)

Both univariate and multifactorial analyses showed that BCLC staging, recurrence months and risk score were significantly associated with patients (P < 0.05) (Fig. 4a and b). The nomogram exhibited exceptional precision in its ability to forecast survival outcomes (Fig. 4c and d).

Next, subgroup analysis was conducted to further confirm the predictive ability of the signature. The results showed a significantly better prognosis in the low-risk group than in the high-risk group in the subgroups of age > / ≤ 50 years, female, male, AFP > / ≤ 300 ng/ml, ALT > / ≤ 50 U/L, tumor size > / ≤ 5 cm, cirrhosis and BCLC stage A (P < 0.0001, P = 0.0017, P = 0.04, P < 0.0001, P = 0.01, P < 0.0001, P = 0.00098, P < 0.0001, P = 0.00023, P = 0.00086, P < 0.0001 and P < 0.0001, respectively) (Fig. 4e–p).

### Potential carcinogenetic mechanisms

**Fig. 5:**
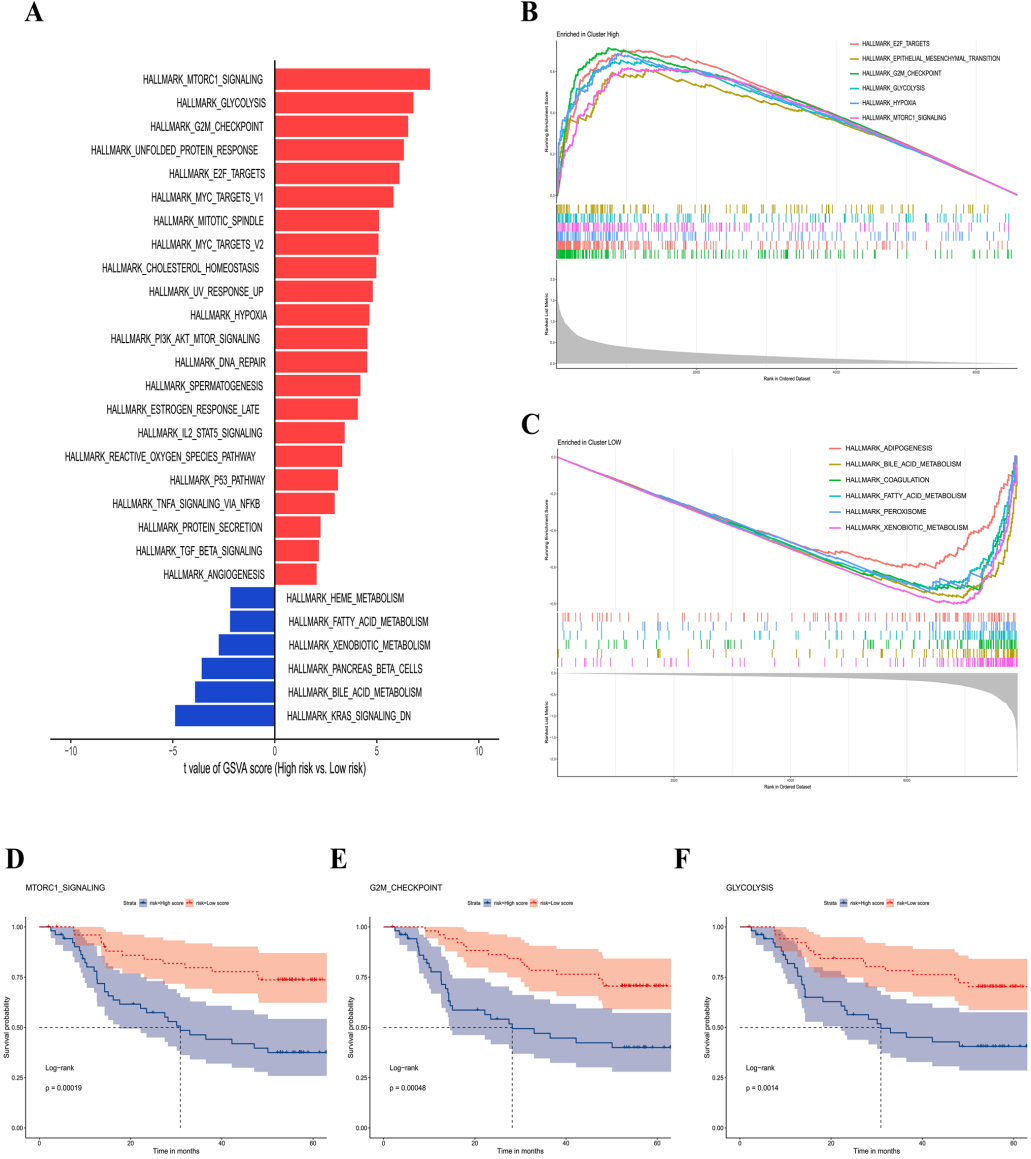
Distinct carcinogenetic mechanisms between the high-risk and low-risk groups. **a** Bar graph for the differential enrichment in carcinogenetic pathways, determined by gene set variation analysis (GSVA), between the high-risk and low-risk groups. Gene set enrichment analysis (GSEA) enrichment plots showing significantly enriched pathways, including (**b**) six upregulated and (**c**) six downregulated pathways. **d–f** KM plots of OS delineating the prognostic landscapes of the three typical oncogenic pathways

Concerning the underlying downstream mechanism between high- and low-risk groups, we performed GSVA and GSEA on the GSE14520 transcriptomic dataset to identify differential cancer hallmark pathways. Based on the GSVA results (Supplementary Table S1), the limma algorithm identified a total of 28 significantly distinct pathways. In the high-risk group, 22 were upregulated and 6 were downregulated. In relation to the 22 enriched pathways depicted in Fig. 5a, the high-risk group was predominantly associated with the activation of carcinogenic pathways, such as MTORC1, GLYCOLYSIS, G2M checkpoint, UNFOLDED_PROTEIN, and E2F. Figure 5a reveals that the high-risk group exhibited metabolic dysregulation, with glycolysis upregulated and oxidative phosphorylation, fatty acid metabolism, and adipogenesis downregulated. In the GSEA, 53 cancer hallmark pathways that were significantly altered were also identified. (Supplemental Table S2) Of these, 29 were upregulated and 24 were downregulated. Consistent with the findings of the GSVA, the high-risk group exhibited predominantly enhanced carcinogenetic pathways and diminished nonglycolysis metabolic pathways (Fig. 5b and c). To determine the prognostic landscape of upregulated hallmark pathways, we drew survival plots for mTORC1, G2M checkpoint, and GLYCOLYSIS, among other significant pathways. As anticipated, the prognosis of HCC patients with these activated pathways was worse (Fig. 5d–f).

### Immune landscape in different risk groups

**Fig. 6:**
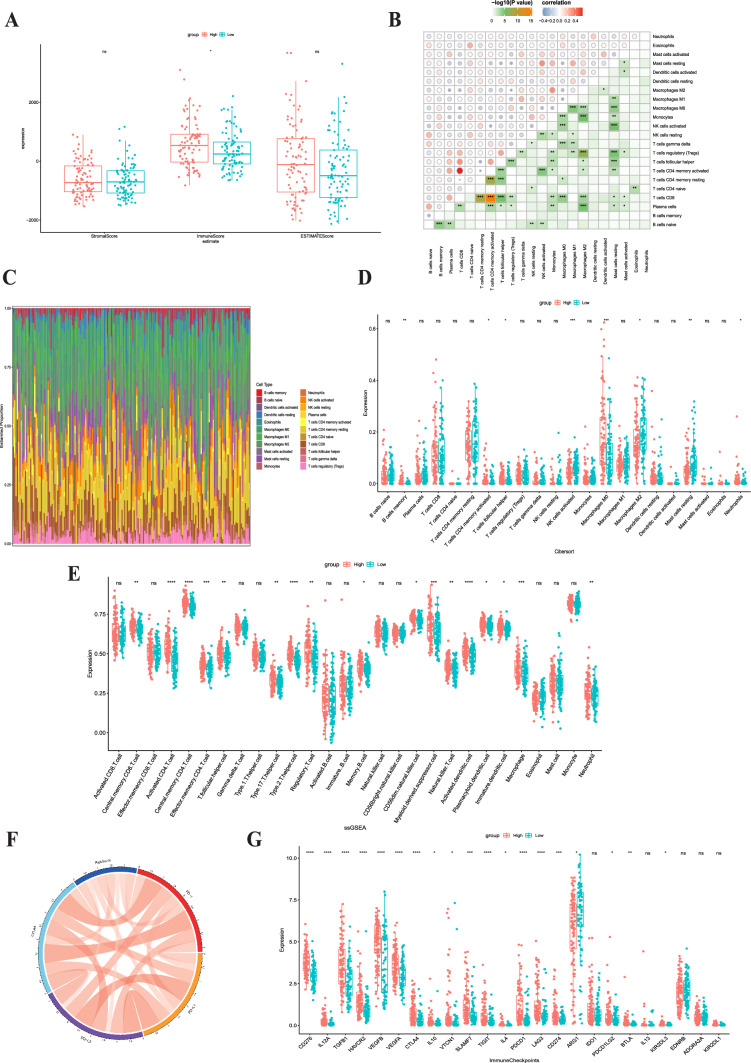
Immune landscape in different risk groups. **a** Stromal, immune, and ESTIMATE scores of the two risk subgroups in the TCGA cohort. **b** Correlation of immunocytes. **c** Immunocyte distribution in the two subgroups. **d, e** The boxplot for the comparison of immunocytes in CIBERSORT and ssGSEA. Chordal graph **f** for the correlation of the common immune checkpoint genes, including PD1, CTLA4, PD-L1, and PD-L2, with the risk score. **g** The correlation of the prognostic signature with 24 immune checkpoint genes

To further investigate the intricate interplay between TACE-induced cellular senescence and tumor immunity, we divided stage II-III liver cancer patients from the TCGA database into high-risk and low-risk groups based on the risk score. The ESTIMATE method was utilized to quantify immune and stromal cell ratios for evaluating TIME and analyzing group differences. We observed that the ImmuneScore was higher in the high-risk group (Fig. 6a). In addition, we utilized CIBERSORT to determine the subtypes of immunocytes in HCC. Figure 6c depicts a remarkable correlation between the proportions of immunocyte subsets (Fig. 6b). We then measured immunocyte concentrations in the two risk groups and discovered that activated NK cells, M2 macrophages, and resting mast cells were more abundant in the low-risk group (Fig. 6d), whereas memory B cells, follicular helper T cells, activated memory CD4 + T cells, M0 macrophages, and eosinophils were more prevalent in the high-risk group. Then, we conducted ssGSEA to delineate the distinct landscape of infiltrated immune cells among the high-risk and low-risk groups (Fig. 6e). The high-risk group had a higher degree of immunosuppressive cells, including myeloid-derived suppressor cells (MDSCs) and regulatory T cells (Fig. 6e). Subsequently, we focused on the analysis of differential expression of immune checkpoint genes between high-risk and low-risk groups. The findings of our study revealed a statistically significant positive correlation between the risk score and the expression levels of PD1, CTLA4, PD-L1, and PD-L2, as seen in Fig. 6f. In addition, substantially elevated expression of immune checkpoint genes that drive T cell functional exhaustion, including PDCD1, CTLA4, LAG3, TIGIT, and HAVCR2, was observed in the high-risk group (Fig. 6g).

### Potential therapeutic value and candidate compound

**Fig. 7:**
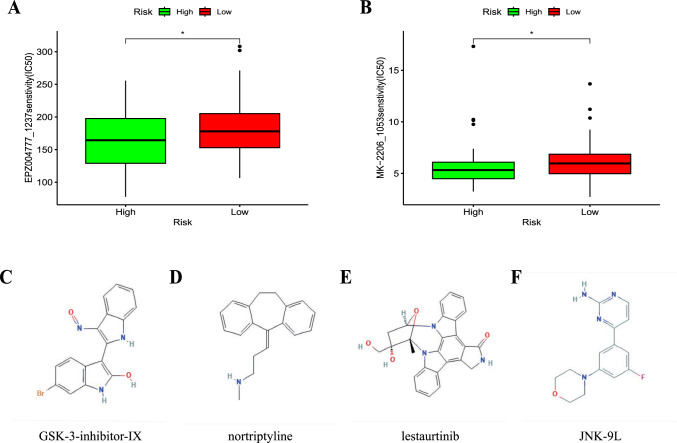
Application of the prognostic signature for drug sensitivity and putative compounds. **a, b** The correlation between the two risk subgroups and drug sensitivity. The 2D structures of the top four potential compounds, GSK-3-inhibitor-IX (**c**), nortriptyline (**d**), lestaurtinib (**e**), and JNK-9L (**f**)

To further determine the potential therapeutic value, we demonstrated the feasibility of identifying sensitive drugs and candidate compounds through the CGP and CMap databases [22, 23]. We found that in the TACE nonresponse group, the chemical agents EPZ004777_1237 and MK-2206_1053 had lower standardized IC50 values, indicating higher efficacy in TACE nonresponse patients (P < 0.05, Fig. 7a and b). Furthermore, based on the results from the HEPG2 cell line, we screened and ranked the top four candidate drugs for TACE nonresponsive patients from a pool of 1796 compounds (GSK-3-inhibitor-IX, nortriptyline, lestaurtinib, and JNK-9L), and their 2D structures are shown in Fig. 7c–f. We also provided information on 25 substitute compounds (Supplementary Table S3), among which 24% (6/25) were CDK inhibitors, indicating their potential efficacy for TACE nonresponsive patients.

## Discussion

HCC is regarded as one of the deadliest cancers with a poor prognosis [24]. Its aggressive growth and late symptomatic presentation often lead to most patients being diagnosed in advanced stages, missing the opportunity for surgical intervention [2]. However, no universally effective treatment exists for advanced-stage HCC; the options are diverse, ranging from immunotherapy and targeted therapy to local treatment [3]. As a primary treatment method for advanced stages, TACE has low initial relief rates, often complicating clinical decision-making. Thus, there is an urgent need to identify the TACE nonresponding subtype via relevant prognostic predictive biomarkers to enhance personalized treatment levels. To date, accumulative evidence has indicated that TACE nonresponsiveness is associated with changes in the TIME, including the exhaustion of CD8 + T cells, proliferation of tumor-associated macrophages, and significant increases in immune checkpoint sites [10, 11]. These changes in the TIME can induce tumor angiogenesis and TACE resistance, leading to tumor recurrence and progression. With advancements in tumor genomics, identifying tumor immune microenvironments based on specific genes, predicting prognosis, determining treatment plans, and selecting drugs have become standardized treatment modes. Cellular senescence can play an indispensable role in the TME via cell-autonomous and paracrine effects, leading to tumor formation and cancer progression [25]. However, the relationship between cellular senescence and TACE nonresponsiveness, the relevant immune checkpoint responses, and the potential for drug intervention remain largely unclear.

In this study, we constructed a risk model for postoperative TACE prognosis based on differentially expressed genes (DEGs) in TACE responders and nonresponders. We found that cellular senescence was significantly enriched in TACE nonresponders compared to responders. Notably, within the PPI network, CDKN2A emerged as a crucial marker of the cellular senescence pathway [26]. This finding further confirms the association between TACE nonresponse and the cellular senescence pathway. Futhermore, we identified nine genes associated with OS probability in GSE14520 in univariate Cox regression analysis. And lasso regression analysis yielded a final prognostic marker comprising four genes: FOXM1, CDK1, CHEK1, and SERPINE1. These four genes have been previously reported to predict classification and prognosis. FOXM1 has been demonstrated to bind to PINT87aa, inducing cellular senescence and promoting hepatocellular carcinoma (HCC) progression [27]. Nevertheless, apart from the high expression of SERPINE1 being associated with poor prognosis after TACE, the influence of the other three genes on TACE nonresponsiveness has not been reported [28, 29]. Furthermore, our risk model demonstrated high specificity for TACE nonresponsiveness and predictive value for clinical prognosis. By elucidating the potential mechanistic distinctions between the high-risk and low-risk groups, we identified the most overactive oncogenic pathways, including glycolysis, the G2M checkpoint, MTORC1, and EMT signaling pathways. Notably, according to the public literature [30–33], all mentioned pathways have been proven to cause immune suppression or weak immunotherapy response.

There is a scarcity of research on whether and how cellular senescence after TACE regulates immune infiltration and immune modulators, thereby affecting the treatment response to immune checkpoint inhibitors. Through ESTIMATE analysis, we found that the TACE nonresponse group had more immune cell infiltration. In further research, we used CIBERSORT and ssGSEA to determine the proportion of immune cells in the two high- and low-risk groups. The results showed that macrophage count and MDSCs were significantly elevated in the high-risk subgroup. Tumor-associated macrophages can secrete a variety of immune suppressive factors and chemokines, decrease antigen presentation, and hinder T-cell function, thereby leading to tumor evasion from immune surveillance [34]. Tumor-associated macrophages can also stimulate fibroblasts to secrete TGF-β signals, leading to the transdifferentiation of fibroblasts into activated myofibroblasts (cancer-associated fibroblasts). These fibroblasts can directly or indirectly regulate the function of effector T cells in the antitumor immune response and contribute to the formation of an immunosuppressive TIME [35]. MDSCs can release SASP-MMPs, promoting tumor cell invasion by directly increasing angiogenesis and lymphangiogenesis [36, 37]. We also found that the predictive model was broadly associated with most of the 50 common immune checkpoint genes, including PD-1, CTLA4, PDL1, and T-cell exhaustion biomarkers, which are related to T-cell-mediated immunotherapy [38]. Overall, this predictive model shows great potential as a comprehensive predictive factor for immune infiltration and evasion as well as the immunotherapy response to immune checkpoint blockade.

We validated the feasibility of identifying sensitive drugs and candidate compounds through the CGP and CMap databases [22, 23].Here, we screened two drugs in the TACE nonresponsiveness, EPZ004777_1237 and MK-2206_1053, revealing their specific therapeutic potential for cell senescence. In addition, we identified the four most promising candidate compounds from a pool of 1796 compounds. Among them, the top-scoring compound, GSK-3-inhibitor-IX, is a glycogen synthase kinase inhibitor. Previous studies have reported that glycogen synthase kinase inhibitors can promote GSK3β phosphorylation, induce cell senescence, and result in tumor suppression in hepatocellular carcinoma [39]. However, other studies have suggested that glycogen synthase kinase inhibitors can inhibit cellular senescence-mediated human fibroblast biomolecular damage, leading to mesenchymal-like fibroblasts becoming more epithelial-like, indicating a mesenchymal-epithelial transition process and exhibiting anti-metastatic activity [40]. We hypothesize that early-stage hepatocellular carcinoma cell senescence may trigger immune activation to eliminate tumor and senescent cells. With the continued action of TACE chemotherapy and the accumulation of senescent hepatocytes and secretion of SASP, the glycogen synthase kinase inhibitor can reverse the EMT process caused by SASP, thereby preventing tumor angiogenesis and tumor recurrence. Therefore, we believe that the specific mechanism of action of GSK-3 inhibitor IX in cell senescence deserves further investigation. Notably, the other three compounds have also been reported to have antitumor capabilities, although their relevance to cellular senescence has yet to be determined [41, 42]. These candidate compounds provide clues for treatment strategies for TACE nonresponsive cases, and further exploration will contribute to a deeper understanding of the interaction mechanisms between these small compounds and cellular senescence.

However, our study has certain limitations. First, there may be biases in the clinical pathological features since the data were retrospectively collected from public sources. Therefore, large-scale prospective studies are needed to confirm our findings. Second, more biological research is required to elucidate the mechanisms and functional differences among different subgroups. Therefore, our subsequent research will focus on investigating the bioinformatics and clinical evidence within this context.

## Conclusions

In summary, we constructed a cellular senescence-related signature that could be used to predict prognosis, immunotherapy response, and potential therapeutic drugs in TACE-treated HCC patients, aiding in the development of personalized treatment plans.

## Methods

### Dataset collection and preprocessing

Genes involved in cellular senescence were selected from the GSE104580 dataset. This dataset consisted of data from 147 patients who underwent TACE as the primary treatment modality for their condition. In this dataset, the assessment of the response to TACE within a 3-month period (after either the first or second TACE session) was conducted by extramural reviewers using the modified Response Evaluation Criteria in Solid Tumors (mRECIST). The main goal of the final group trial was tumor response. The patients who achieved a complete response or a partial response were defined as the TACE responders. Conversely, patients who had stable disease or progressive disease were defined as the TACE nonresponders [43]. RNA extracted from tumor biopsies of liver cancer patients who received TACE treatment was analyzed using Affymetrix gene arrays (Affymetrix, Santa Clara, CA, USA).

In the GSE14520 development cohort, we included 74 patients who received adjuvant TACE treatment, which refers to the use of TACE as an additional therapy following surgical resection of the tumor to target any residual disease, and 30 patients who received TACE treatment after recurrence. Additionally, we included 85 patients who underwent liver resection alone [44]. Similarly, a total of 54 patients who underwent TACE with complete survival time and status data from the International Cancer Genome Consortium (ICGC, https://dcc.icgc.org/releases/current/Projects/LIRI-JP) were employed as the validation cohort. For The Cancer Genome Atlas (TCGA, http://cancergenome.nih.gov), a total of 177 HCC patients were included for immune-related analysis.

### Establishment of a potential prognostic cellular senescence-related gene signature

The limma package(v3.45.2) [45] in R software v4.3.0 (R Foundation for Statistical Computing, Vienna, Austria) normalized gene expression data by formula log2 to compute DEGs between the TACE response and nonresponse groups in GSE104580 under the following conditions: P < 0.05, |log2fold change (FC)|> 0.5. The clusterprofiler package [46] in R was used to analyze the dysregulated genes in the TACE nonresponse group using KEGG. Twenty genes enriched in cellular senescence were selected and then analyzed by univariate regression in GSE14520. Finally, we screened nine survival-related genes: cyclin-dependent kinase 4 (CDK4), forkhead box M1 (FOXM1), cyclin B2 (CCNB2), C-X-C motif chemokine ligand 8 (CXCL8), cyclin-dependent kinase 1 (CDK1), cell division cycle 25A (CDC25A), cyclin B1 (CCNB1), checkpoint kinase 1 (CHEK1), and serpin family E member 1 (SERPINE1). The protein‒protein interaction network (PPI) was constructed using the data retrieved from the Search Tool for the Retrieval of Interacting Genes/Proteins (STRING) and visualized using Cytoscape v3.9.1. (https://cytoscape.org/). OS and RFS were calculated using the Kaplan–Meier (K–M) method; The Kaplan–Meier (K–M) method was employed to compute OS and RFS. Statistical analysis was conducted using the survival package, while visualization was performed using the survminer tool. Using the glmnet program, we conducted LASSO regression and developed a prognostic signature to reduce the size of certain regression coefficients while retaining the advantages of subset reduction [47, 48]. Consequently, we established the following formula to calculate the risk score:

$$Risk\,score = \sum\limits_{i = 1}^{n} {Expi \times coefi}$$

where n represents the number of genes in the signature and β represents the coefficient of genes obtained from LASSO regression.

### Nomogram construction

For evaluation of individual patients, a nomogram was constructed based on risk score, sex, age, multinodular status, cirrhosis, TNM stage and BCLC stage. The composite nomogram scores derived from the aggregation of all variables for each patient may be utilized to predict the rates of survival at 1, 3, and 5 years.

### Functional enrichment analysis

The study utilized gene set enrichment analysis (GSEA) to investigate the underlying mechanism of TACE nonresponse. This analysis focused on the 50 hallmark pathways (v7.5.1) available in the molecular signature database. The results were computed using the “clusterProfiler” package and visualized using the “enrichplot” package [49, 50].

The GSVA enrichment score for each patient was calculated using the “GSVA” package [51] for the aforementioned 50 oncogenic pathways. Subsequently, Kaplan–Meier (KM) diagrams were generated in order to ascertain the prognostic pattern of the most prominent overlapping oncogenic pathways identified using GSVA and GSEA.

### Immune cell infiltration and immunotherapy response

The study employed ESTIMATE, CIBERSORT, and single-sample gene set enrichment analysis (ssGSEA) techniques to assess and quantify the disparity in the TIME between high- and low-risk groups. The measurement of immunological checkpoints, immune cell infiltration, and immune inhibitors (or stimulators) in different risk scores of HCC patients was conducted in light of the potential use of immunotherapy.

### Potential antitumor drug sensitivity and candidate compound

Drug response information, measured with AUC across various cancer cells, of 368 drugs was obtained from the Cancer Genome Project (CGP) via the R package “Oncepredict” [22]. Each TACE sample’s normalized half-maximal inhibitory concentrations (IC50) were quantified. A boxplot was generated to capture the differential drug sensitivity between the high- and low-risk groups.

To determine the putative drug for the TACE nonresponse group, we performed chemotherapeutic forecasting via the “query” module of the connectivity map (CMap, https://clue.io/query) [23]. After the upregulated and downregulated genes were uploaded and analyzed for the high- and low-risk groups, permuted data were generated. To gain more insight, the 2D drug structures of the top four candidate compounds were visualized using the PubChem website (https://pubchem.ncbi.nlm.nih.gov/).

### Statistical analysis

All statistical analyses were conducted using R software v4.3.0 (R Foundation for Statistical Computing, Vienna, Austria) and relevant packages. All statistical analyses were two-tailed, and P < 0.05 were considered statistically significant. Univariate and multivariate Cox regression models were used to validate characteristics useful for predicting TACE-treated patients’ prognosis. We used a t-test to evaluate the differences between the two groups. With ggplot2, a correlation matrix was constructed.

### Supplementary Information

**Additional file 1: Table S1.** The GSVA results regarding the risk groups (High vs. Low).

**Additional file 2: Table S2.** The GSEA results regarding the risk groups (High vs. Low).

**Additional file 3: Table S3.** The top 25 potential therapeutic compounds.

## Data Availability

The transcriptome in this research is available in the GEO database (https://www.ncbi.nlm.nih.gov/geo/) under accession numbers GSE14520 and GSE104580, TACE database (http://www.cancergenome.nih.gov) and ICGC database (http://www.dcc.icgc.org).

## Abbreviations

HCC: Hepatocellular carcinoma
TACE: Transarterial chemoembolization
KEGG: Kyoto Encyclopedia of Genes and Genomes
GSVA: The gene set variation analysis
GSEA: Gene set enrichment analysis
TIME: Tumor immune microenvironment
TCGA: The Cancer Genome Atlas
ICGC: The International Cancer Genome Consortium
