# Supplementary material for: A cellular senescence-related signature for predicting prognosis, immunotherapy response, and candidate drugs in patients treated with transarterial chemoembolization (TACE)
Source: Discov Oncol. 2024 Jul 8;15:271. doi: 10.1007/s12672-024-01116-8 (PMC11231123; doi:10.1007/s12672-024-01116-8)
Supplement: Supplementary file 1 — Additional file 1: Table S1. The GSVA results regarding the risk groups (High vs. Low). [file 12672_2024_1116_MOESM1_ESM.docx]

**Table S1. The GSVA results regarding the risk groups (High vs. Low)**

| Pathways | t-value | *P*-value | Adjust p-value |
| --- | --- | --- | --- |
| MTORC1_SIGNALING | 7.607704 | 8.04E-14 | 4.02E-12 |
| GLYCOLYSIS | 6.797334 | 2.12E-11 | 5.29E-10 |
| G2M_CHECKPOINT | 6.531953 | 1.17E-10 | 1.95E-09 |
| UNFOLDED_PROTEIN_RESPONSE | 6.326109 | 4.23E-10 | 5.29E-09 |
| E2F_TARGETS | 6.113116 | 1.54E-09 | 1.54E-08 |
| MYC_TARGETS_V1 | 5.818907 | 8.65E-09 | 7.20E-08 |
| MITOTIC_SPINDLE | 5.106939 | 4.12E-07 | 2.89E-06 |
| MYC_TARGETS_V2 | 5.084183 | 4.63E-07 | 2.89E-06 |
| CHOLESTEROL_HOMEOSTASIS | 4.975739 | 8.00E-07 | 4.44E-06 |
| UV_RESPONSE_UP | 4.802832 | 1.88E-06 | 8.53E-06 |
| HYPOXIA | 4.63762 | 4.13E-06 | 1.72E-05 |
| PI3K_AKT_MTOR_SIGNALING | 4.540626 | 6.49E-06 | 2.41E-05 |
| DNA_REPAIR | 4.532394 | 6.75E-06 | 2.41E-05 |
| SPERMATOGENESIS | 4.196283 | 3.03E-05 | 0.000101 |
| ESTROGEN_RESPONSE_LATE | 4.068442 | 5.21E-05 | 0.000163 |
| IL2_STAT5_SIGNALING | 3.424731 | 0.000648 | 0.001704 |
| REACTIVE_OXYGEN_SPECIES_PATHWAY | 3.303996 | 0.000997 | 0.002492 |
| P53_PATHWAY | 3.093552 | 0.002048 | 0.004877 |
| TNFA_SIGNALING_VIA_NFKB | 2.931905 | 0.003467 | 0.007881 |
| PROTEIN_SECRETION | 2.241095 | 0.0253 | 0.052709 |
| TGF_BETA_SIGNALING | 2.160612 | 0.031029 | 0.057461 |
| ANGIOGENESIS | 2.05147 | 0.040555 | 0.072419 |
| HEME_METABOLISM | -2.17586 | 0.029865 | 0.057433 |
| FATTY_ACID_METABOLISM | -2.19104 | 0.028744 | 0.057433 |
| XENOBIOTIC_METABOLISM | -2.74571 | 0.006177 | 0.013428 |
| PANCREAS_BETA_CELLS | -3.58067 | 0.000364 | 0.001011 |
| BILE_ACID_METABOLISM | -3.90826 | 0.000101 | 0.000297 |
| KRAS_SIGNALING_DN | -4.88499 | 1.26E-06 | 6.28E-06 |
