# Supplementary material for: A cellular senescence-related signature for predicting prognosis, immunotherapy response, and candidate drugs in patients treated with transarterial chemoembolization (TACE)
Source: Discov Oncol. 2024 Jul 8;15:271. doi: 10.1007/s12672-024-01116-8 (PMC11231123; doi:10.1007/s12672-024-01116-8)
Supplement: Supplementary file 2 — Additional file 2: Table S2. The GSEA results regarding the risk groups (High vs. Low). [file 12672_2024_1116_MOESM2_ESM.docx]

**Table S2. The GSEA results regarding the risk groups (High vs. Low)**

| Pathways | NES | P-value | Q-value |
| --- | --- | --- | --- |
| G2M_CHECKPOINT | 1.857921 | 1E-10 | 5.53E-10 |
| E2F_TARGETS | 1.814031 | 1E-10 | 5.53E-10 |
| ANGIOGENESIS | 1.706721 | 9.45E-05 | 0.000174 |
| HYPOXIA | 1.66855 | 1E-10 | 5.53E-10 |
| GLYCOLYSIS | 1.610413 | 6.69E-10 | 2.96E-09 |
| MTORC1_SIGNALING | 1.587825 | 1E-10 | 5.53E-10 |
| CHOLESTEROL_HOMEOSTASIS | 1.584994 | 2.44E-05 | 4.91E-05 |
| EPITHELIAL_MESENCHYMAL_TRANSITION | 1.567337 | 2.04E-08 | 7.51E-08 |
| MITOTIC_SPINDLE | 1.520553 | 2.48E-07 | 6.85E-07 |
| IL2_STAT5_SIGNALING | 1.504299 | 7.02E-06 | 1.72E-05 |
| FATTY_ACID_METABOLISM | 1.497211 | 0.000217 | 0.000345 |
| ESTROGEN_RESPONSE_LATE | 1.469452 | 2.25E-05 | 4.91E-05 |
| XENOBIOTIC_METABOLISM | 1.457862 | 0.000248 | 0.000366 |
| MYC_TARGETS_V1 | 1.445395 | 8.13E-08 | 2.57E-07 |
| ESTROGEN_RESPONSE_EARLY | 1.404669 | 0.000219 | 0.000345 |
| COMPLEMENT | 1.392195 | 0.000637 | 0.000828 |
| ANDROGEN_RESPONSE | 1.389238 | 0.002756 | 0.003207 |
| KRAS_SIGNALING_UP | 1.37702 | 0.000481 | 0.000664 |
| SPERMATOGENESIS | 1.367836 | 0.006953 | 0.006986 |
| MYOGENESIS | 1.333336 | 0.00673 | 0.006986 |
| TNFA_SIGNALING_VIA_NFKB | 1.329282 | 0.00093 | 0.001142 |
| COAGULATION | 1.315697 | 0.014423 | 0.012753 |
| P53_PATHWAY | 1.309025 | 0.003163 | 0.003496 |
| MYC_TARGETS_V2 | 1.296774 | 0.021178 | 0.017338 |
| INFLAMMATORY_RESPONSE | 1.280944 | 0.010686 | 0.010271 |
| UNFOLDED_PROTEIN_RESPONSE | 1.262987 | 0.018474 | 0.015707 |
| UV_RESPONSE_UP | 1.25687 | 0.01248 | 0.011494 |
| PI3K_AKT_MTOR_SIGNALING | 1.250481 | 0.026973 | 0.021294 |
| UV_RESPONSE_DN | 1.222862 | 0.042957 | 0.032744 |
| ESTROGEN_RESPONSE_LATE | -1.23275 | 0.038961 | 0.04272 |
| P53_PATHWAY | -1.26825 | 0.034112 | 0.03903 |
| KRAS_SIGNALING_UP | -1.32282 | 0.003311 | 0.006176 |
| EPITHELIAL_MESENCHYMAL_TRANSITION | -1.33169 | 0.007399 | 0.010817 |
| OXIDATIVE_PHOSPHORYLATION | -1.3356 | 0.003755 | 0.006176 |
| APOPTOSIS | -1.35108 | 0.010088 | 0.013274 |
| HEME_METABOLISM | -1.37001 | 0.000399 | 0.001049 |
| HYPOXIA | -1.37235 | 0.003607 | 0.006176 |
| ANDROGEN_RESPONSE | -1.38857 | 0.007996 | 0.011075 |
| INTERFERON_GAMMA_RESPONSE | -1.39508 | 0.000554 | 0.001326 |
| UV_RESPONSE_DN | -1.4142 | 0.001555 | 0.003411 |
| UV_RESPONSE_UP | -1.42744 | 0.00209 | 0.004232 |
| ANGIOGENESIS | -1.42749 | 0.030488 | 0.036469 |
| ESTROGEN_RESPONSE_EARLY | -1.43891 | 7.73E-05 | 0.000254 |
| ADIPOGENESIS | -1.45511 | 4.18E-05 | 0.000183 |
| MTORC1_SIGNALING | -1.47106 | 0.006284 | 0.009727 |
| COMPLEMENT | -1.47234 | 4.85E-05 | 0.000183 |
| GLYCOLYSIS | -1.49633 | 0.000194 | 0.000567 |
| REACTIVE_OXYGEN_SPECIES_PATHWAY | -1.51972 | 0.012264 | 0.015369 |
| PEROXISOME | -1.70474 | 1.21E-07 | 6.38E-07 |
| FATTY_ACID_METABOLISM | -1.71606 | 1.54E-10 | 1.01E-09 |
| COAGULATION | -1.75184 | 1E-10 | 8.77E-10 |
| BILE_ACID_METABOLISM | -1.8115 | 1E-10 | 8.77E-10 |
| XENOBIOTIC_METABOLISM | -1.91777 | 1E-10 | 8.77E-10 |
