# Supplementary material for: A cellular senescence-related signature for predicting prognosis, immunotherapy response, and candidate drugs in patients treated with transarterial chemoembolization (TACE)
Source: Discov Oncol. 2024 Jul 8;15:271. doi: 10.1007/s12672-024-01116-8 (PMC11231123; doi:10.1007/s12672-024-01116-8)
Supplement: Supplementary file 3 — Additional file 3: Table S3. The top 25 potential therapeutic compounds. [file 12672_2024_1116_MOESM3_ESM.docx]

**Table S3. The top 25 potential therapeutic compounds**

| Score | Compound name | Description |
| --- | --- | --- |
| -99.73 | GSK-3-inhibitor-IX | Glycogen synthase kinase inhibitor |
| -99.7 | nortriptyline | Tricyclic antidepressant |
| -99.69 | lestaurtinib | FLT3 inhibitor |
| -99.62 | JNK-9L | JNK inhibitor |
| -99.5 | triptolide | RNA polymerase inhibitor |
| -99.47 | RS-17053 | Adrenergic receptor antagonist |
| -99.47 | CGP-60474 | CDK inhibitor |
| -99.46 | ZG-10 | JNK inhibitor |
| -99.4 | PIK-75 | DNA protein kinase inhibitor |
| -99.37 | cyclopamine | Smoothened receptor antagonist |
| -99.32 | PHA-793887 | CDK inhibitor |
| -99.29 | alvocidib | CDK inhibitor |
| -99.24 | thiostrepton | FOXM1 inhibitor |
| -99.21 | JAK3-inhibitor-VI | JAK inhibitor |
| -99.18 | JNJ-7706621 | CDK inhibitor |
| -99.09 | chromomycin-a3 | DNA binding agent |
| -99.04 | flupentixol | Dopamine receptor antagonist |
| -98.94 | A-443644 | AKT inhibitor |
| -98.87 | bisindolylmaleimide-ix | CDK inhibitor |
| -98.86 | camptothecin | Topoisomerase inhibitor |
| -98.75 | BH3I-1 | BCL inhibitor |
| -98.69 | BMS-345541 | IKK inhibitor |
| -98.5 | palbociclib | CDK inhibitor |
| -98.37 | SN-38 | Topoisomerase inhibitor |
| -98.34 | aminopurvalanol-a | Tyrosine kinase inhibitor |
